# Laparoscopic versus open liver resection for intrahepatic cholangiocarcinoma: a systematic review of propensity score-matched studies

**DOI:** 10.1007/s13304-023-01648-8

**Published:** 2023-11-02

**Authors:** Ya-Fei Hu, Hai-Jie Hu, Wen-Jie Ma, Yan-Wen Jin, Fu-Yu Li

**Affiliations:** Department of Biliary Surgery, West China Hospital of Sichuan University, Chengdu, 610041 Sichuan China

**Keywords:** Intrahepatic cholangiocarcinoma (ICCA), Laparoscopic liver resection (LLR), Open liver resection (OLR), Propensity score-matched studies (PSM)

## Abstract

Outcomes of laparoscopic liver resection (LLR) versus open LR (OLR) for intrahepatic cholangiocarcinoma (ICCA) are heterogeneous. We aimed to compare LLR and OLR for ICCA based on propensity-score-matched (PSM) studies. Two reviewers independently searched the online databases (PubMed, Embase, and Cochrane Library) for PSM studies that compared LLR and OLR for ICCA. The Ottawa–Newcastle Quality Assessment Scale with a cutoff of ≥ 7 was used to define higher-quality literature. Only ‘high-quality’ PSM analyses of the English language that met all our inclusion criteria were considered. A total of ten PSM trials were included in the analyses. Compared with OLR, although the lymph node dissection (LND) (RR = 0.67) and major hepatectomy rates were lower in the LLR group (RR = 0.87), higher R0 resections (RR = 1.05) and lower major complications (Clavien–Dindo grade ≥ III) (RR = 0.72) were also observed in the LLR group. In addition, patients in the LLR group showed less estimated blood loss (MD = − 185.52 ml) and shorter hospital stays as well (MD = − 2.75 days). Further analysis found the overall survival (OS) (HR = 0.91), disease-free survival (DFS) (HR = 0.95), and recurrence-free survival (HR = 0.80) for patients with ICCA after LLR were all comparable to those of OLR. LLR for selected ICCA patients may be technically safe and feasible, providing short-term benefits and achieving oncological efficacy without compromising the long-term survival of the patients.

**Supplementary Information:**

The online version contains supplementary material available at 10.1007/s13304-023-01648-8.

## Introduction

Intrahepatic cholangiocarcinoma (ICCA) and hepatocellular carcinoma (HCC) are the most seen primary liver cancers (PLC). Studies reported most PLCs were HCC (> 80%), and ICCA only accounted for 10–15% [1]. Compared with HCC, ICCA is rarer, and there is a lack of large population-based or randomized-controlled studies (RCT) of ICCA treatment. Reviewing existing literature for evidence of ICCA treatment is required [2–5].

There are significant differences between HCC and ICCA [6] when regarding the patient's basic demographic characteristics and tumor biological features. Patients with ICCA usually had a worse prognosis, with 5-year overall survival (5-OS) being 20–40% versus that of 50–70% for patients with HCC. Studies have also found that ICCA patients tend to present a more advanced tumor burden compared to HCC, including a higher rate of lymph node involvement (27.6% vs. 7.7%) and larger tumor size (proportion of tumors ≥ 5 cm (63.7% vs. 46.3%)) [1]. Therefore, although laparoscopic liver resection (LLR) is gaining momentum for HCC [1, 7, 8], evidence of LLR application for ICCA has not been quantified in the aggregate [9–13].

Due to inferior tumor biological behaviors, the difficulty of hilar lymph node dissection (LND), and hepato-biliary reconstruction, LLR application for ICCA is in the initial phase and mainly reserved in high-volume experienced medical centers. Most existing studies including meta-analyses (MAs) had reported LLR for selected ICCA patients was technically safe and feasible, with comparable oncological outcomes to the open LR (OLR) [10, 14, 15]. Contradictory results were still reported from several studies [16].The safety, feasibility, and oncological efficiency of LLR for ICCA are related to ICCA tumor stages and the surgeons’ experience. Therefore, significant heterogeneity is presented in published studies. Moreover, postoperative complications and survival outcomes after either LLR or OLR may vary with the availability of efficacious antitumor therapies and improved perioperative management over time. Thus, published systematic reviews need to be updated with high-quality propensity-score-matched (PSM) studies.

Based on the reasons above, we aimed to compare both perioperative-related and long-term survival outcomes of LLR and OLR in patients with ICCA through a meta-analytic approach. Only recently published PSM studies of high quality were included in analyses.

## Materials and methods

### Literature search

The protocol for this study had been registered at PROSPERO (CRD42022348032). Our paper followed the Preferred Reporting Items for Systematic Reviews and Meta-Analyses (PRISMA) 2020 [17].

Two reviewers (*Author 1 and Author 2*) systematically searched online databases including PubMed, Cochrane Library, and Embase from data inception to May 12, 2023, to find studies that might be suitable for our meta-analyses. The search strategy was based on the PICOS principle, combining MESH and their associated/related terms. The search items were presented as follows: ‘Intrahepatic cholangiocarcinoma [MeSH Terms]’ and ‘open liver resection [MeSH Terms]’ and ‘laparoscopic liver resection [MeSH Terms]’ and ‘propensity score-matched analyses [MeSH Terms]’ or ‘open liver surgery’ or ‘open hepatectomy’ or ‘minimally invasive liver resection’ or ‘minimally invasive hepatectomy’ or ‘laparoscopic hepatectomy’ or ‘PSM analyses’ or ‘matched studies’ or ‘ICCA’ or ‘Intrahepatic cholangiocarcinomas’. More detailed search terms are presented in Supplementary Table S3.

The entire texts of possibly qualifying papers were reviewed by the same 2 reviewers (*Author 1 and Author 2*) separately. A third reviewer (*Corresponding Author*) was consulted to settle any disagreements met. To avoid literature that might be ignored during our search, we reviewed the citations of retrieved eligible articles as well as the conference proceedings manually. Besides, to avoid missing studies that were published after our first search, we performed re-research before the submission on June 15, 2023.

### Inclusion criteria

Studies that were eligible for the meta-analyses should meet the following criteria:

i. PSM studies of the English language.
ii. All including patients were pathologically diagnosed with ICCA after postoperative pathological diagnosis.
iii. Use of LLR or OLR for curative-intent resection of ICCA.
iv. Comparing LLR versus OLR for ICCA with sufficient data including perioperative results, and long-term oncological outcomes.
v. Full text available.

Papers were excluded if they were met any of the following:

- Non-English languages.
- Non-comparative analyses, animal, or laboratory studies.
- Abstracts only, meetings, books, or Letters to the Editors.
- Studies that lack adequate clinical data.
- Studies included patients with combined pathologies besides ICCA such as ICCA combined with hepatocellular carcinoma or metastatic liver diseases were excluded. Studies reported patients with perihilar, distal cholangiocarcinoma, and gallbladder cancer were also not considered.
- Studies of low quality were also excluded from the analyses.

### Quality assessments

The quality of the included studies was assessed independently at both the individual study level and outcome level by two reviewers. The Newcastle–Ottawa Scale (NOS) [18] was used to evaluate the risk of bias of each included PSM study The NOS assessment criteria give a maximum of 9 points for risk of bias in three areas: Selection of the cohort, Comparability of exposed and nonexposed participants, and Assessment of results. Literature with a score of ≥ 7 was considered to have a low risk of bias and then was classified as high quality. (Supplementary Table S2).

### Data extraction

Data extraction was conducted independently by 2 reviewers *(Author 1 and Author 2***)**, with any conflicts referred to a third reviewer **(***Corresponding Author***)** for clarification.

We gathered data from the included PSM studies. Study characteristics including but not limited to author/year of publication, research duration, study type (multicenter/single center and retrospective/prospective), and study population were extracted and are presented in Table 1. The patients’ basic demographic features and tumor characteristics in each group (LLR and OLR) such as patients' median age, gender, presence of liver cirrhosis, tumors histology, tumor number, and median diameter can be found in Supplementary Table S1.

**Table 1:** Characteristics of included studies

Author/year	Study type	Data origin/country	Inclusion time	Definition of LND	Definition of Major LR	LLR in total/after PSM	OLR in total/after PSM	Main conclusions	NOS score
Brustia/2022	Multi-Retro	Three independent multicenter databases/France	2000–2018	LN removal of station 12 /8/13. For left-sided tumors, station 7 and1 should also be considered	LR involving 3 or more contiguous Couinaud’s segments	146/89	709/89	Survival advantage of LLR versus OLR was observed for ICCA; LLR for ICCA seems feasible with similar rates of morbidity, compared with OLR	9
Hobeika/2021	Multi-Retro	Multiple HPB centers /France	2000–2017	Any LN harvest from hepatoduodenal ligament to the retro pancreatic and common hepatic artery area	LR involving 3 or more contiguous Couinaud’s segments	127/109	421/109	laparoscopic approach did not substantially improve quality of care of patients with resect able ICCA	8
Ratti/2020	Multi-Retro	Two European referral centers	2004–2017	Formal LND was a complete LN removal of station 8/12 and not performing LND when cirrhosis presence	Left/right or central hepatectomies	114/104	209/104	Feasibility, safety, and oncological efficiency of the laparoscopic approach in the management of ICCA	9
Ratti/2021	Single-Retro	Milan	2004–2020	Formal LND was a complete LN removal of station 8/12 and not performing LND when cirrhosis presence	LR involving 3 or more contiguous Couinaud’s segments	179/150	267/150	MIS for ICCA had advantages in perioperative outcomes and oncological non-inferior to open counterpart. OS and DFS were found to be similar between 2 approaches	8
Kang/2020	Single-Retro	Seoul	2004–2015	Metastatic LN were found/NA	LR of > 3 segments or resection of the right posterior segments	30/24	61/24	LLR for ICCA is technically feasible and safe, providing short-term benefits without increasing complications or affecting long-term survival	8
Zhu/2019	Single-Retro	China	2012–2017	LND performed only when an enlarged LN around the hepatoduodenal ligament was detected, wide lymphadenectomy was avoided	LS of > 3 segments or resection of the right posterior segments	20/18	63/36	LLR for large or multiple ICCA could obtain similar short/long-term outcomes compared to OLR, and LND was also feasible during LLR	8
Sahakyan/2023	multicenter-Retro	Four European expert centers	2012–2019	NA	LR of ≥ 3 consecutive liver segments	50/50	86/50	Laparoscopic resection seems to be associated with improved short-term and with similar long-term outcomes compared with open surgery in patients with ICCA	8
Shen/2023	Single-Retro	China	2010–2021	NA	NA	127/70	43/35	Compared with ICCA treated by OLR,the LLR group obtained superior perioperative period outcomes. LLR could enable ICC patients to receive an equivalent long-term prognosis compared to OLR	8
Yang/2022	Multi-Retro	Three independent multicenter databases in China	2011–2018	NA	LR of ≥ 3 segments or involving the posterior superior segments	150/122	645/122	laparoscopic treatment for early ICC may have certain advantages based on the long-term results; ICCA patients treated with laparoscopy seemed to have better short-term outcomes	8
Salehi/2022	Multi-Retro	NCDB databases/USA	2010–2016	NA	NA	140/115	848/115	MILR is associated with worse lymphadenectomy and survival in patients with ICCA greater than 4 cm requiring major hepatectomy	9

*LND* Lymph node dissection, *muti-retro/single-retro* multicenter/single center retrospective studies, *LS/LR* liver surgery/liver resection, *HPB* hepato-pancreato-biliary, *LR* liver resection, *ICCA* intrahepatic cholangiocarcinoma, *LLR* laparoscopic liver resection, *OLR* open liver resection, *MILR* minimally invasive liver resection, *NA* not applicable

### Definitions

To help comprehensive and accurate measurement of complications that occurred after LLR and OLR, only studies that reported complications graded according to the Clavien–Dindo classification were considered for analyses. Major complications were defined as Clavien–Dindo grade III/IV in the included studies. Textbook Outcome (TO) [12] was a new composite-outcome index. The included 2 studies both considered patients with negative margins (R0), without transfusion, no severe surgical complications, prolonged hospital stay, readmissions, and no postoperative mortality as to have a TO.

### Statistical analyses

The primary objective of our meta-analysis was to assess the safety, feasibility, and long-term outcomes (OS, DFS, and RFS) for resected ICCA patients after LLR and OLR. The Risk ratios (RRs) were used to assess binary variables and mean differences (MDs) to assess continuous variables. The hazard ratio (HR) for survival outcomes was calculated using the statistical method of Tierney et al. If the pooled HR and its 95%CI (confidence interval) overlapped 1, LLR had statistically comparable survival effects to OLR.

Heterogeneity between studies was assessed using Cochran’s *Q* test and Higgins *I*^2^ statistic. *I*^2^ values of 25%, 50%, and 75% represented low, moderate, and high levels of heterogeneity, respectively. An *I*^2^ greater than 50% (*I*^2^ > 50%) was defined as the criterion for significant heterogeneity. Random-effect models were used if significant heterogeneity emerged after the analyses. Only if the analyses showed low and/or moderate heterogeneity (*I*^2^ ≤ 50%) were fixed-effects models considered. Sensitivity analysis using the leave-one-out method was performed to assess the robustness of the results for study results with significant heterogeneity.

Potential publication bias was statistically assessed using Egger’s linear regression. A *P* value of greater than 0.05 for Egger’s test indicated the absence of significant statistical publication bias. To determine the effect of individual studies on the pooled estimates, sensitivity analyses were performed by serially excluding each study. Statistical significance was defined as two-tailed *P* values less than 0.05.

All statistical analyses were performed using RevMan software 5.3 (The Cochrane Collaboration, Oxford, UK) and R software version 4.0.0 (R Foundation for Statistical Computing, Vienna, Austria), and the Meta package was used for data analysis.

## Results

### Study characteristics

**Fig. 1:**
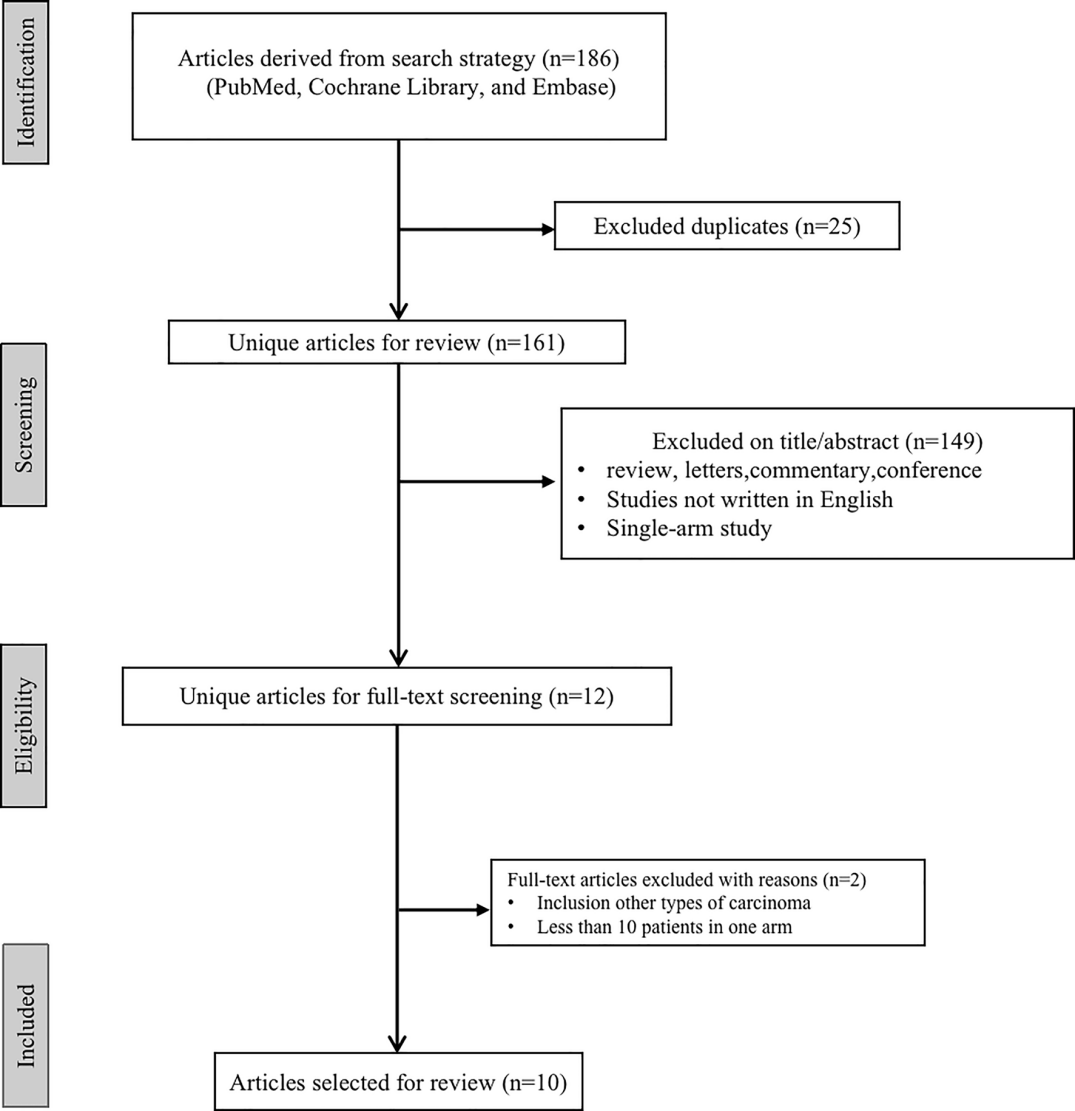
The PRISMA selection flow diagram

After comprehensive database searching and removing duplicates, 186 studies were originally identified. After a screening of titles and abstracts, 164 were removed for reasons of non-English language, single-arm analyses, case reports, reviews, letters, commentary, and conferences. Twelve studies were further evaluated for full text. Ten studies that met all inclusion criteria were finally included in the meta-analysis [16, 19–27]. The flow diagram of the literature inclusion is presented in Fig. 1.

All the included studies were retrospective. According to NOS, included literature was of high quality, with 3 studies obtaining a score of 9, and 7 having a score of 8. We included 2 studies by Ratti et al. [19, 20] for the patient population in these studies were from different regions (Milan and Two European referral centers), and the research duration was also different. We summarized the baseline characteristics of the included studies in Table 1. The main outcomes of the meta-analyses are presented in Table 2. 

Although all included analyses were after the PSM method, given the differences in sample inclusion criteria among literature, we still compared whether important tumor characteristics differed significantly between the two groups (LLR and OLR). These indicators have been considered key factors that may influence the prognosis for ICCA patients in the previously published literature. There were no statistically significant differences in the basic demographics and tumor characteristics between the laparoscopic and open arms. Differences were noted in terms of patients in the LLR group having smaller tumor size versus OLR with SMD = − 0.13 (95% CI − 0.23 to − 0.03; *P* = 0.02; *I*^2^ = 0%). Both positive LN status and multiple tumor numbers (≥ 2) in the LLR group were comparable to OLR, with RR = 0.68 (95% CI 0.44–1.06, *P* = 0.09; *I*^2^ = 70%) and RR = 0.95; (95%CI: 0.76–1.20; *P* = 0.68; *I*^2^ = 0%), respectively. Significant heterogeneity was seen in the comparison of LN status in LLR versus OLR (*I*^2^ = 70%).

**Table 2:** Main outcomes of the meta-analyses

Short-term outcomes	Variable names	Included studies	Participants	Statistical method	Effect estimate
Perioperative outcomes	Major hepatectomy	9	1445	Risk Ratio (M–H, Fixed, 95% CI)	0.87 [0.78, 0.97]
	R0 resection	7	1293	Risk Ratio (M–H, Fixed, 95% CI)	1.05 [1.01, 1.09]
	LND rate	7	1106	Risk Ratio (M–H, Random, 95% CI)	0.67 [0.49, 0.91]
	Major complications rates (Clavien–Dindo grade ≥ III)	9	1455	Risk Ratio (M–H, Fixed, 95% CI)	0.72 [0.56, 0.94]
	90-day mortality	8	1532	Risk Ratio (M–H, Fixed, 95% CI)	0.69 [0.46, 1.03]
	Textbook outcomes (TO)	2	396	Risk Ratio (M–H, Fixed, 95% CI)	1.42 [1.05, 1.92]
	Retrieved lymph nodes (LN)	3	726	Mean Difference (IV, Random, 95% CI)	0.24 [-1.46, 1.94]
	Liver failure after surgery	4	780	Risk Ratio (M–H, Fixed, 95% CI)	0.64 [0.32, 1.28]
	Duration of hospital stay (days)	9	1507	Mean Difference (IV, Random, 95% CI)	-2.75 [-3.78, -1.71]
	The conversion rate of LLR	7	1163	67/590	11.35%
	Lymphatic fistula rates	2	508	Risk Ratio (M–H, Fixed, 95% CI)	0.29 [0.11, 0.79]
	Biliary leakage rate	4	667	Risk Ratio (M–H, Fixed, 95% CI)	0.55 [0.28, 1.08]
	Blood loss (ml)	6	933	Mean Difference (IV, Fixed, 95% CI)	-185.52 [-216.11, -156.92]
	Perioperative blood transfusion rate	8	1407	Risk Ratio (M–H, Fixed, 95% CI)	0.45 [0.34, 0.59]
	Duration of surgery (min)	8	1277	Mean Difference (IV, Random, 95% CI)	10.53 [-13.75, 34.81]
Postoperative Long-term results	RFS (Recurrence-free survival)	3	740	Hazard Ratio (IV, Fixed, 95% CI)	0.80 [0.63, 1.02]
	OS (Overall survival)	9		Hazard Ratio (IV, Fixed, 95% CI)	0.91 [0.81, 1.03]
	DFS (Disease-free survival)	5		Hazard Ratio (IV, Fixed, 95% CI)	0.95 [0.80, 1.14]
Tumor characteristics	Tumor size (cm)	9	1467	Std. Mean Difference (IV, Fixed, 95% CI)	-0.13 [-0.23, -0.03]
	Positive LN status	9	1455	Risk Ratio (M–H, Random, 95% CI)	0.68 [0.44, 1.06]
	Multiple tumor numbers (≥ 2)	8	1237	Risk Ratio (M–H, Fixed, 95% CI)	0.95 [0.76, 1.20]

### Perioperative outcomes

**Fig. 2:**
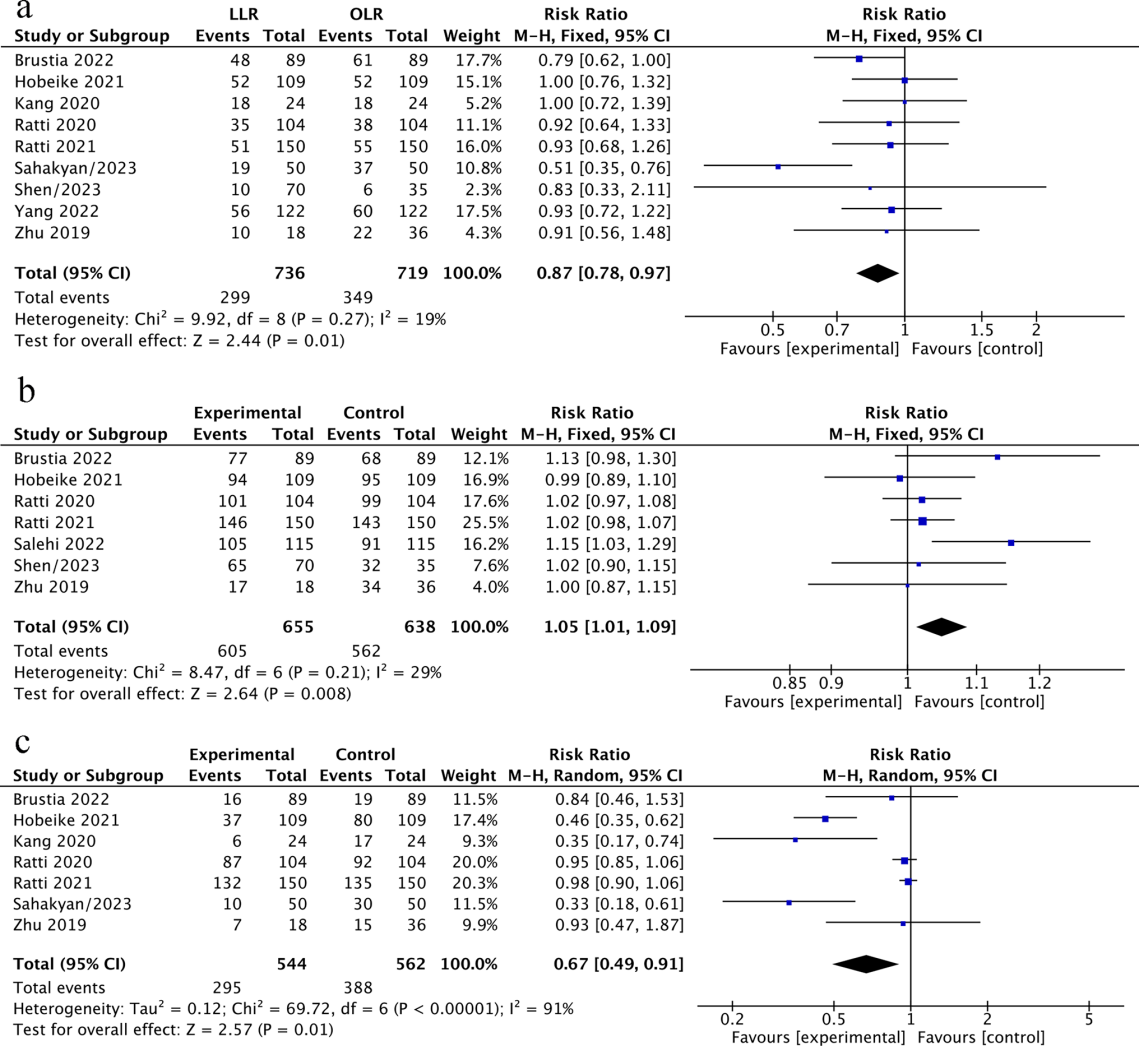
**a**–**c** Operative outcomes of LLR versus OLR. **a** Major hepatectomy rates. **b** R0 resection. **c** LN dissection rate

With a pooled RR = 0.87 (95%CI 0.78–0.97, *P* = 0.01; *I*^2^ = 19%), we found that the incidence of major hepatectomy was lower in LLR than in OLR (Fig. 2a). Regarding LN dissection (LND) rates, we also found that patients after LLR group had lower LND rates than those in the OLR group (RR = 0.67; 95% CI 0.49–0.91; *P* = 0.01). However, there was a significant degree of heterogeneity among the included studies with *I*^2^ = 91% (Fig. 2c). Although the major hepatectomy and LND rates were lower in the LLR group, we found patients in the LLR group achieved a higher incidence of R0 resection than those in the OLR group (RR = 1.05, 95%CI 1.01–1.09, *P* = 0.008; *I*^2^ = 29%) (Fig. 2b).

**Fig. 3:**
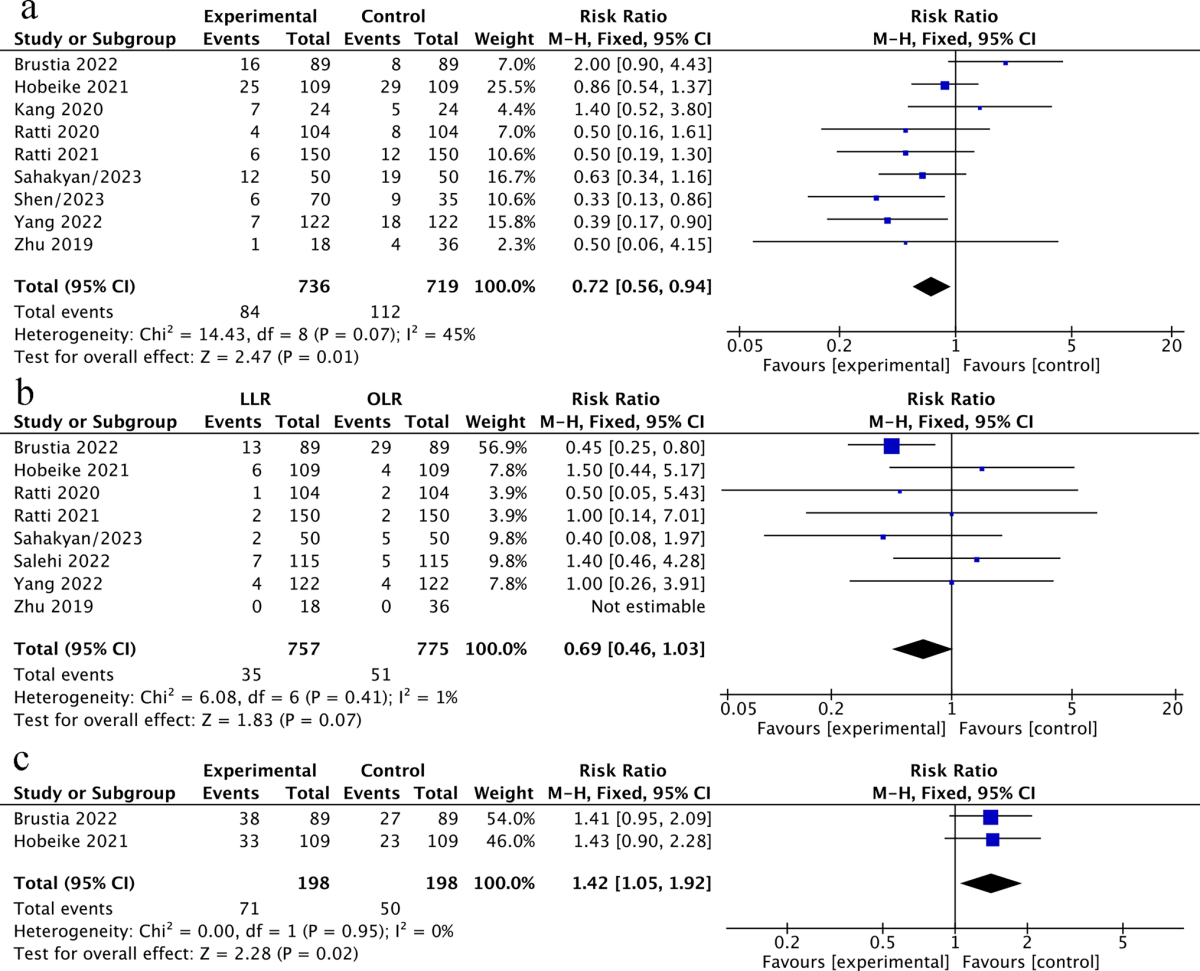
**a**–**c** Perioperative outcomes of LLR versus OLR. **a** Major complication rates (Clavien–Dindo grade ≥ 3). **b** The 90-day mortality rates. **c** Textbook outcomes (TO)

We also determined the postoperative outcomes for patients with ICCA after LLR and OLR. Our analyses showed that compared with the OLR group, the major complications (Clavien–Dindo grade ≥ III) rate was lower for ICCA patients after LLR with RR = 0.72 (95%CI 0.56–0.94, *P* = 0.01; *I*^2^ = 45%) (Fig. 3a). The 90-day mortality rate was comparable between the two arms with a pooled RR = 0.69 (95%CI 0.46–1.03, *P* = 0.07; *I*^2^ = 1%) (Fig. 3b). Textbook outcome (TO), a novel composite measure for the surgical care quality, which represents the ideal hospitalization for patients with ICCA undergoing curative-intent resection. To compare the difference in TO between LLR and OLR, we included 2 studies with the same definitions of TO. In the end, we found that the patients in the LLR group achieved a higher TO than the patients after OLR (RR = 1.42; 95%CI: 1.05–1.92, *P* = 0.02). No heterogeneity was found in the analyses with *I*^2^ = 0% (Fig. 3c).

To assess the value of using LLR for ICCA more comprehensively, we have summarized other perioperative outcomes as follows: LLR resulted in lower blood loss (MD = − 185.82 ml), shorter hospital stays (MD = − 2.75 days), less lymphatic fistula incidence (RR = 0.29), and lower perioperative blood transfusion rate (RR = 0.45) than the OLR group. In addition, there was no significant heterogeneity in most of the outcomes. Nevertheless, the number of LNs harvested (MD = 0.24), the duration of surgery (MD = 10.53 min), the incidence of liver failure (RR = 0.64), and the bile leakage (RR = 0.55) rate for the patients in the LLR group were comparable to patients after OLR. The pooled results are shown in Supplementary Appendix: Figure S1, Figure S2. Finally, the conversion rate of laparoscopic procedures was reported in 7 studies and ranged between 0% and 18.0%, which is slightly lower than the results in other published literature (7.4–20%).

### Postoperative long-term outcomes

**Fig. 4:**
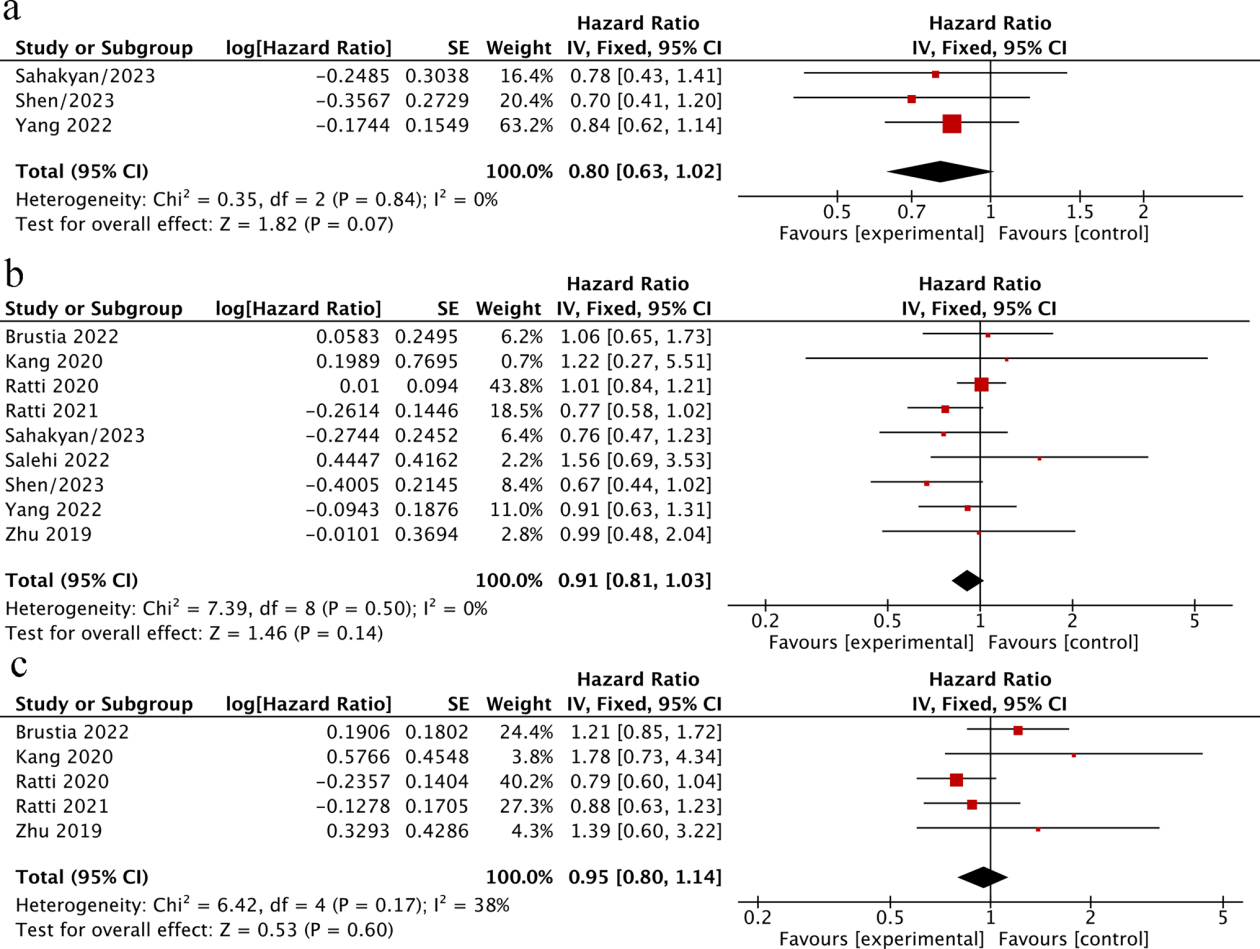
**a**–**c** Postoperative long-term outcomes of LLR versus OLR. **a** Recurrence-free survival for ICCA patients after LLR versus OLR. **b** Overall Survival for ICCA patients after LLR versus OLR. **c** Disease-free survival for ICCA patients after LLR versus OLR

Regarding the recurrence-free survival (RFS) in ICCA patients after surgery, no difference was found between LLR and OLR. Patients in the LLR group had comparable RFS compared to those with OLR (HR = 0.80, 95%CI 0.63–1.02, *P* = 0.07; *I*^2^ = 0%. Figure 4a). No significant difference was also resulted between LLR and OLR regarding either overall survival (OS) (HR = 0.91; 95% CI 0.81–1.03, *P* = 0.14; *I*^2^ = 0%; Fig. 4b) or disease-free survival (DFS) (HR = 0.95, 95% CI 0.80–1.14; *P* = 0.60; *I*^2^ = 38%; Fig. 4c), and there was no significant heterogeneity among the studies (*I*^2^ = 0% and 38%, respectively).

### Sensitivity analyses and publication bias

Results with significant heterogeneity were pooled with the random-effect model and performed with sensitivity analyses. In our study, substantial heterogeneity was observed in the analyses of the hospital stay, surgery duration, and LND rate. After performing the sensitivity analysis using the leave-one-out method, we found no changes in the risk estimate or the level of significance in terms of these outcomes. The significant heterogeneity among the included studies may be related to the surgeon's varied experiences, different hospital volumes, and LND indications. For the assessment of publication bias, we performed Egger's test and found no significant bias in terms of length of hospital stay (*P* = 0.2168), surgery duration (*P* = 0.4558), and LND rate (*P* = 0.0652) (Supplementary Figure S2a–c). However, due to the limited study samples (*N* = 3), the publication bias for retrieved lymph nodes number objectively exists.

## Discussions

For patients with ICCA, radical surgery resection for a negative margin (R0) is the only curative treatment [28, 29]. Due to the technical difficulty and uncertainty in long-term efficacy, most of the ICCA surgeries are performed under an open surgery method. LLR for ICCA is just starting, and uncertainty that exists over the subject of ICCA can be routinely solved through an LLR procedure. Only limited high-volume centers had demonstrated the feasibility and safety of LLR for ICCA. LLR technique for ICCA remained challenging at most medical centers, particularly when dealing with large (> 5 cm), multiple (> 2), or advanced ICCA tumors [11, 13, 19, 30, 31].

Published studies included different stages of ICCA in LLR and OLR; thus, the baseline characteristics of the patients were not totally matched [20, 21]. After analyzing, we found in most of the current literature, ICCA patients treated with the laparoscopy method usually had tumors of early stages and smaller sizes; thus, there is less need for large-scale hepatectomy or LND. Owing to the earlier staging of ICCA within the LLR group, previous research has not definitively established the advantages of LLR for ICCA, when compared with the OLR arm [32, 33]. Therefore, in our study, we only included PSM studies for the comparison of LLR versus OLR.

Previous studies had reported some factors including R0 resections, large tumor size, and positive LN status may influence the OS of ICCA patients. This interaction on the hand demonstrated that LLR should be performed in selected ICCA with the feasibility of adequate tumor resection and LND [20, 31, 33]. Our study found that LLR for some selected ICCA cases resulted in similar oncological and long-term survival outcomes to those of OLR, with the advantages of less blood loss, major complication rate, and shorter hospital stays. Prior studies have also shown that LLR could confer better short-term perioperative outcomes to OLR with comparable long-term survival prognosis [14, 15]. Due to smaller incisions, both wound complication rate and postoperative pain were significantly decreased in the LLR group which also help to improve the patient’s recovery. Regarding similar oncological outcomes including R0 resection and major hepatectomy rate to OLR, LLR for some selected ICCA seemed to be the optimal method.

However, LLR was associated with inferior LND in most of the published studies [14, 15] which made the effectiveness of laparoscopic LND remained controversial [32]. Martin et al. showed the laparoscopic LND rate was significantly lower than that of open surgery (39% vs. 61%, *P* < 0.01); while other scholars believed that the magnifying effect of laparoscopy was helpful to identify LN for a comprehensive surgery resection. Ratti et al. showed that laparoscopic LND could result in a similar number of LN to open surgery with lower complications incidence related to LND. According to the guidelines, LND was acknowledged as a standard treatment for ICCA; those conflicting published results suggested that LLR for ICCA currently may not be totally optimal [16, 19, 34].

A meta-analysis conducted by Zhou et al. suggested that LND had a limited impact on the ICCA patient's long-term prognosis, besides a higher incidence of postoperative complications was seen in the LND group. In fact, the significance of LND in ICCA was to obtain more accurate pathological staging for guiding further adjuvant treatments. Therefore, experts from the American Hepatobiliary and Pancreatic Association recommend routine LND in the ICCA treatment and suggested that at least 6 lymph nodes should be obtained after surgical resection for tumor staging. LND was to obtain adequate numbers of LN, merely comparing the LND rate in OLR and LLR may not be as important as the compassion for the average number of retrieved LN in LLR and OLR. In our analyses, we resulted in lower LND rates for ICCA after LLR compared with OLR, while in further analyses, we demonstrated a comparable average number of retrieved LN in patients with LLR and OLR. Therefore, we may conclude that LLR for selected ICCA could be practiced at experienced centers with strict ongoing practice evaluation, and the oncological efficacy was not inferior to patients with OLR.

Although more and more medical centers have carried out LLR for ICCA, we should still pay attention to the surgical indications and strictly control the quality of the surgery. The objective of surgery is to ensure R0 resection and improve patients’ survival [35–39]. We still need large-scale multicenter studies to explore the safety, feasibility, surgery indications, contraindications, and long-term efficacy of LLR application in ICCA [12, 31, 32, 40–44]

### Strengths and limitations

Our MAs provide an updated and thorough comparison of perioperative and long-term survival outcomes for ICCA patients following LLR and OLR. However, there are several limitations to our study. First, selection bias is inherent in our included retrospective studies, and publication bias is also unavoidable due to the limited number of studies in our MAs. Besides, although we only included studies of PSM methods, other features such as tumor stages or biology behaviors may not be strictly comparable in LLR and OLR, which may confound our results. Furthermore, given that only retrospective studies were available for our MAs, it may cause a higher level of heterogeneity. In addition, there are limited studies in our MAs, and the meta-regression for some baseline characteristics on patients’ survival is not actionable, reducing our MAs’ statistical power. Finally, data from Africa and Asia were scarce, which highlighted the need for more multicenter studies.

## Conclusions

LLR for selected ICCA patients may be technically safe and feasible, providing short-term benefits and achieving oncological efficacy without compromising the long-term survival of the patients. Although an increasing number of medical centers have been performing LLR for ICCA, the procedure is still in the initial phase of exploration. More evidence is needed to validate the use of LLR for ICCA.

### Supplementary Information

Below is the link to the electronic supplementary material.

Supplementary file1 (DOCX 22 KB). Supplementary Table S1. Patients’ basic demographics and tumor characteristics after PSM analyses. Supplementary Table S2. The Newcastle Ottawa Scale (NOS) of included studies. Supplementary Table S3. Detailed Search strategies.

Supplementary file2 (DOCX 12995 KB). Supplementary Fig S1a. Retrieved lymph nodes (LN) of LLR versus OLR. b. Liver failure after LLR versus OLR. c. Duration of hospital stay (days) of LLR versus OLR. d. Lymphatic fistula rates of LLR versus OLR. e. Biliary leakage rate of LLR versus OLR. f. Blood loss (ml) of LLR versus OLR. g. Perioperative blood transfusion rate of LLR versus OLR. h. Duration of surgery (min) of LLR versus OLR. Supplementary Fig S2a-c. Publication bias of length of hospital stay. a. Publication bias of length of hospital stay. b. Publication bias of length of surgery duration. c. Publication bias LND rate.

## Data Availability

All data generated or analyzed during this study are included in the published article.

## Abbreviations

ICCA: Intrahepatic cholangiocarcinoma
LLR: Laparoscopic liver resection
OLR: Open liver resection
PSM: Propensity score matching
MAs: Meta-analyses
LND: Lymph node dissection
PLC: Primary liver cancer
RCT: Randomized-controlled studies
TO: Textbook outcome
