# Supplementary material for: Laparoscopic versus open liver resection for intrahepatic cholangiocarcinoma: a systematic review of propensity score-matched studies
Source: Updates Surg. 2023 Nov 2;75(8):2049–61. doi: 10.1007/s13304-023-01648-8 (PMC10710389; doi:10.1007/s13304-023-01648-8)
Supplement: Supplementary file 1 — Supplementary file1 (DOCX 22 KB). Supplementary Table S1. Patients’ basic demographics and tumor characteristics after PSM analyses. Supplementary Table S2. The Newcastle Ottawa Scale (NOS) of included studies. Supplementary Table S3. Detailed Search strategies. [file 13304_2023_1648_MOESM1_ESM.docx]

**Supplementary Table S1. Patients’ basic demographics and tumor characteristics after PSM analyses**

| **STable 1. Patients’ basic demographics and tumor characteristics after PSM analyses** | | | | | | | | |
| --- | --- | --- | --- | --- | --- | --- | --- | --- |
| **Author/year** | **Age (years)^a^** | | **Male sex** | | **BMI^a^** | | **Liver Cirrhosis (Grade), N** | |
|  | LLR | OLR | LLR | OLR | LLR | OLR | LLR | OLR |
| Brustia/2022 | 65.24±11.4 | 67.92±8.97 | 52 | 38 | 25.82± 4.55 | 25.28± 4.40 | 21(F3–F4) | 6(F3–F4) |
| Jinhuan/2022 | NA | NA | 68 | 73 | 23.6(21.7-25.8) | 24.2(21.4-27.2) | NA | NA |
| Salehi/2022 | 65.7(34–87) | 67.1(40–85) | NA | NA | NA | NA | NA | NA |
| Hobeika/2021 | 67 (60–72) | 61 (52–68) | NA | NA | 25.8(23.0-29.3) | 25.8(22.9-29.3) | 30(F3–F4) | 21(F3-F4) |
| Ratti/2021 | 59±5 | 61±6 | 70 | 68 | 24.1±1.9 | 24.6±1.6 | 13 | 13 |
| Ratti/2020 | 61 ± 4 | 62 ± 7 | 92 | 86 | 24.6(2.6) | 24.9(1.8) | 20 | 13 |
| Kang/2020 | 66.8(9.7) | 68.1(10.2) | 15 | 15 | NA | NA | 3 | 3 |
| Zhu/2019 | 54.1(16.6) | 55.6(9.8) | 10 | 19 | 23.0(3.4) | 23.4(5.2) | 6 | 15 |
| Sahakyan/2023 | 65.5±9.2 | 65.4±10.6 | 23 | 22 | 28.3±4.2 | 27.2±4.8 | NA | NA |
| Shen/2023 | 66 (59–71) | 66 (59–73) | 35 | 17 | 23.0 (20.9–25.3) | 21.8 (20.1–24.5) | 8 | 6 |
| **Author/year** | **Mean tumor size (cm)**  **(Mean(range)/ mean± SD)** | | **Multiple Tumor Number** | | **Differentiation (poor)** | | **Positive LN status, N** | |
|  | **LLR** | **OLR** | **LLR** | **OLR** | **LLR** | **OLR** | **LLR** | **OLR** |
| Brustia/2022 | 4.67±2.56 | 5.32±3.73 | NA | NA | 12 | 14 | 6 | 17 |
| Jinhuan/2022 | 4.35(3.00-6.00) | 5.00(3.50-6.00) | 21 | 19 | 60 | 56 | 5 | 7 |
| Salehi/2022 | 4.2 (3.0–6.0) | 4.4(3.0–7.0) | NA | NA | 28 | 35 | NA | NA |
| Hobeika/2021 | NA | NA | NA | NA | NA | NA | 7 | 30 |
| Ratti/2021 | 5.3±2.3 | 5.8±1.2 | 20 | 21 | 25 | 16 | 56 | 53 |
| Ratti/2020 | 3.9±1.7 | 4.1±1.2 | 15 | 15 | 17 | 14 | 32 | 31 |
| Kang/2020 | 4.1±1.8) | 4.7±3.3 | 2 | 2 | NA | NA | 8 | 2 |
| Zhu/2019 | 6(3-9) | 6(4-9) | 4 | 8 | 12 | 27 | 3 | 9 |
| Sahakyan/2023 | 5.51 (3.15) | 5.81 (2.92) | 28 | 28 | 14 | 9 | 2 | 10 |
| Shen/2023 | 4.6 (3.9–6.1) | 5.4 (3.1–7.5) | 7 | 6 | NA | NA | 17 | 11 |
| lymph node dissection (LND); LR: liver resection; Intrahepatic cholangiocarcinoma (ICCA); Laparoscopic liver resection (LLR); Open liver resection (OLR); NA: not applicable; **^a^**: data is presented as (Mean(range)/mean± SD); lymph node dissection (LND); LR: liver resection; Intrahepatic cholangiocarcinoma (ICCA); Laparoscopic liver resection (LLR); Open liver resection (OLR); NA: not applicable. | | | | | | | | |

**Supplementary Table S2. The Newcastle Ottawa Scale (NOS) of included studies.**

| **Author/year** | Selection | Comparability | Exposure/outcomes | Overall score |
| --- | --- | --- | --- | --- |
| Brustia/2022 | 4 | 2 | 3 | 9 |
| Jinhuan/2022 | 4 | 2 | 2 | 8 |
| Salehi/2022 | 4 | 2 | 3 | 9 |
| Hobeika/2021 | 4 | 2 | 2 | 8 |
| Ratti/2021 | 4 | 2 | 3 | 9 |
| Ratti/2020 | 4 | 1 | 3 | 8 |
| Kang/2020 | 4 | 2 | 2 | 8 |
| Zhu/2019 | 4 | 1 | 3 | 8 |
| Sahakyan/2023 | 4 | 1 | 3 | 8 |
| Shen/2023 | 4 | 1 | 3 | 8 |

**Supplementary Table S3. Detailed Search strategies**

| **Detailed Search strategies** |
| --- |
| Intrahepatic cholangiocarcinoma [MeSH Terms]: "cholangiocarcinoma"[MeSH Terms]  Laparoscopic: "laparoscopes"[MeSH Terms] OR "laparoscopes"[All Fields] OR "laparoscope"[All Fields] OR "laparoscopic"[All Fields] OR "laparoscopically"[All Fields] OR "laparoscopies"[All Fields] OR "laparoscopy"[MeSH Terms] OR "laparoscopy"[All Fields] OR "laparoscopic"[All Fields]  liver resection [MeSH Terms]: "hepatectomy"[MeSH Terms]  liver resection: "hepatectomy"[MeSH Terms] OR "hepatectomy"[All Fields] OR ("liver"[All Fields] AND "resection"[All Fields]) OR "liver resection"[All Fields]  Propensity Score: "propensity score"[MeSH Terms] OR ("propensity"[All Fields] AND "score"[All Fields]) OR "propensity score"[All Fields]  Matching: "match"[All Fields] OR "matched"[All Fields] OR "matches"[All Fields] OR "matching"[All Fields] OR "matchings"[All Fields]  liver: "liver"[MeSH Terms] OR "liver"[All Fields] OR "livers"[All Fields] OR "liver's"[All Fields]  Filters: Humans, English。 |
