# Supplementary material for: Laparoscopic versus open liver resection for intrahepatic cholangiocarcinoma: a systematic review of propensity score-matched studies
Source: Updates Surg. 2023 Nov 2;75(8):2049–61. doi: 10.1007/s13304-023-01648-8 (PMC10710389; doi:10.1007/s13304-023-01648-8)

Supplementary Fig S1a. Retrieved lymph nodes (LN) of LLR versus OLR

Supplementary Fig S1b. Liver failure after LLR versus OLR

Supplementary Fig S1c. Duration of hospital stay (days) of LLR versus OLR

Supplementary Fig S1d. Lymphatic fistula rates of LLR versus OLR

Supplementary Fig S1e. Biliary leakage rate of LLR versus OLR

Supplementary Fig S1f. Blood loss (ml) of LLR versus OLR

Supplementary Fig S1g. Perioperative blood transfusion rate of LLR versus OLR

Supplementary Fig S1h. Duration of surgery (min) of LLR versus OLR


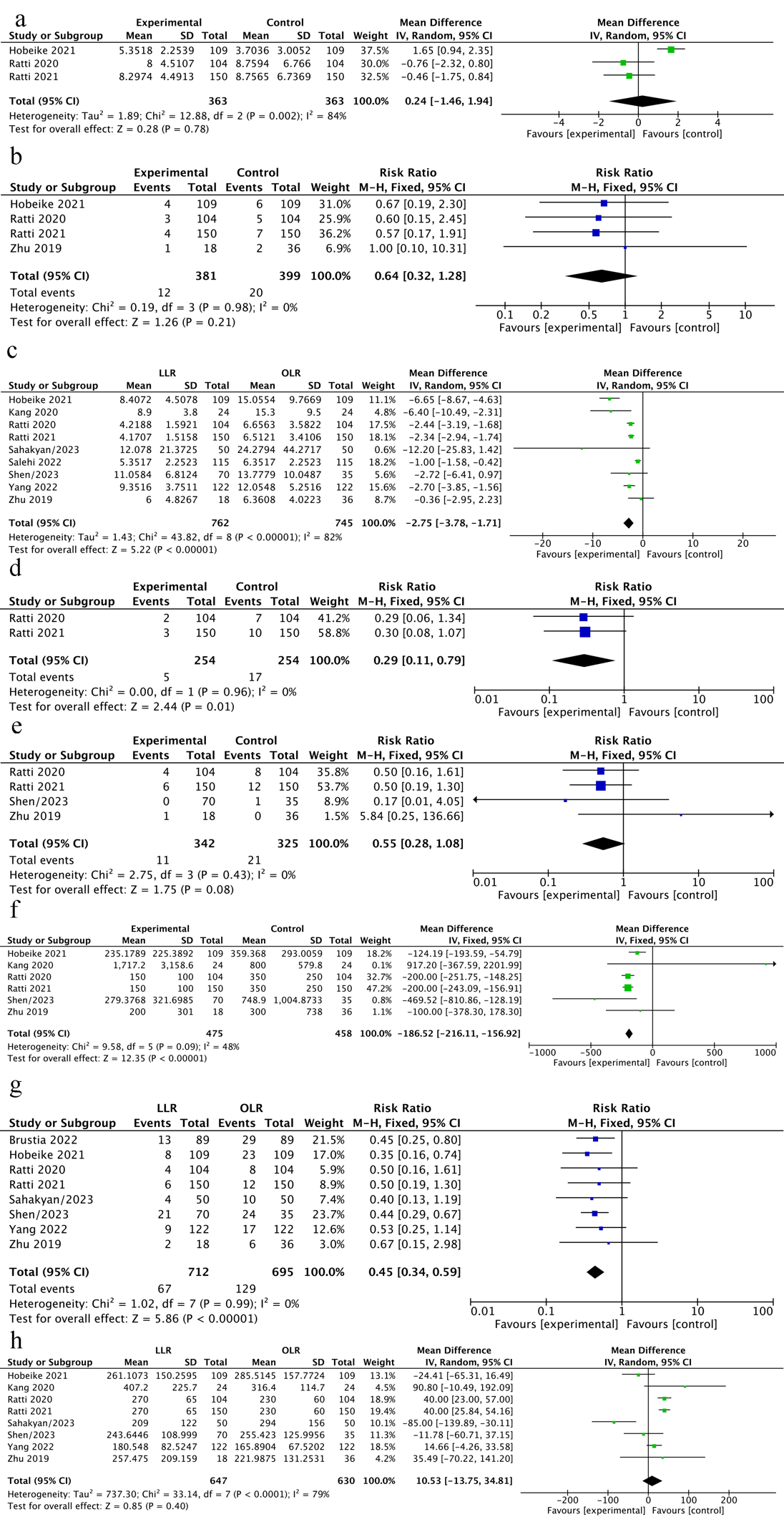


Supplementary Fig S2a-c. Publication bias of length of hospital stay.

Supplementary Fig S2a. Publication bias of length of hospital stay.

Supplementary Fig S2b. Publication bias of length of surgery duration

Supplementary Fig S2c. Publication bias LND rate


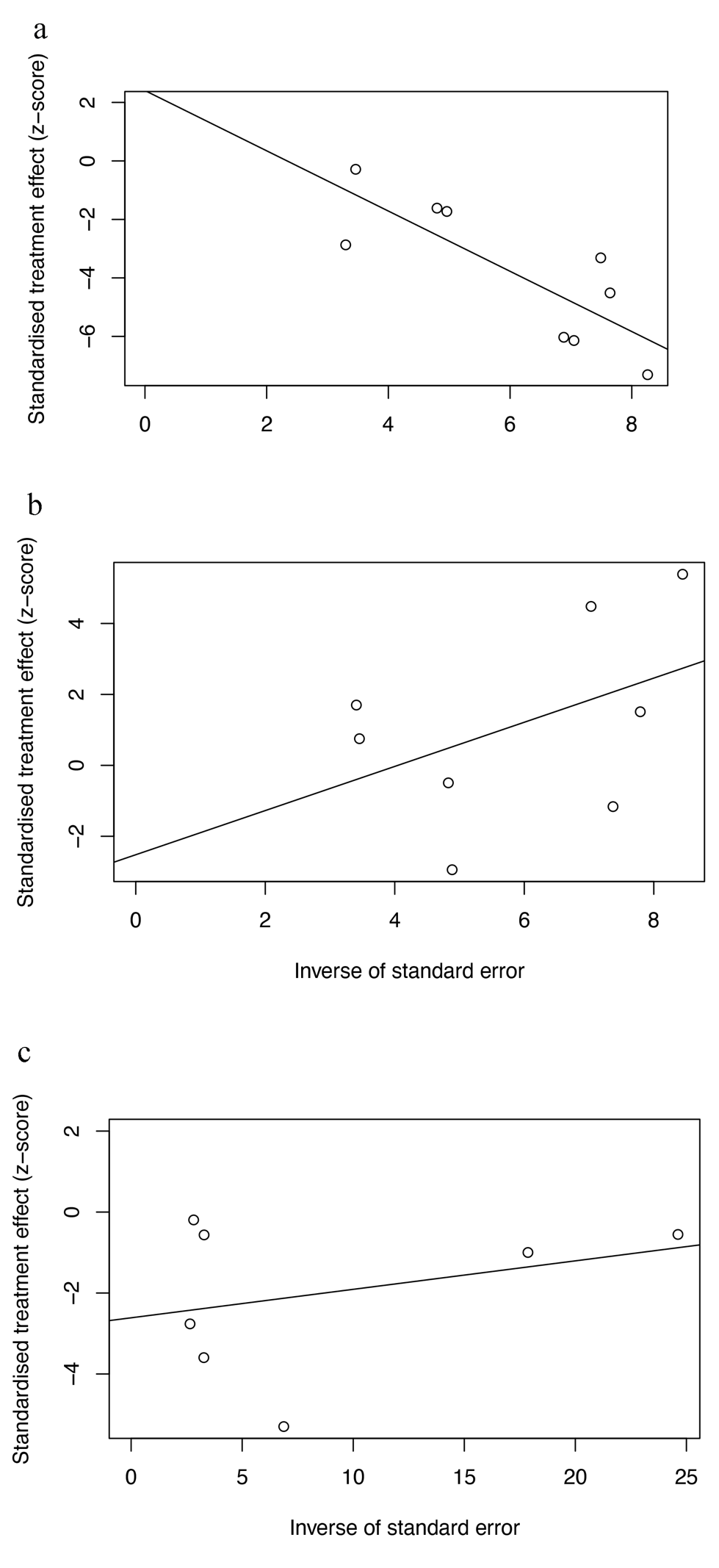

Supplement: Supplementary file 2 — Supplementary file2 (DOCX 12995 KB). Supplementary Fig S1a. Retrieved lymph nodes (LN) of LLR versus OLR. b. Liver failure after LLR versus OLR. c. Duration of hospital stay (days) of LLR versus OLR. d. Lymphatic fistula rates of LLR versus OLR. e. Biliary leakage rate of LLR versus OLR. f. Blood loss (ml) of LLR versus OLR. g. Perioperative blood transfusion rate of LLR versus OLR. h. Duration of surgery (min) of LLR versus OLR. Supplementary Fig S2a-c. Publication bias of length of hospital stay. a. Publication bias of length of hospital stay. b. Publication bias of length of surgery duration. c. Publication bias LND rate. [file 13304_2023_1648_MOESM2_ESM.docx]
